# Magnitude of childhood immunization and its associated factors in Sub-Saharan Africa: A mixed effect count regression analysis

**DOI:** 10.1371/journal.pone.0354918

**Published:** 2026-08-26

**Authors:** Setegn Muche Fenta, Haile Mekonnen Fenta, Hailegebrael Birhan Biresaw, Seyifemickael Amare Yilema, Denekew Bitew Belay, Maru Mekie, Minilik Derseh, Astewle Andargie Baye, Yegnanew Shiferaw, Ding Geng Chen

**Affiliations:** 1 Department of Statistics, College of Natural and Computational Sciences, Debre Tabor University, Debre Tabor, Ethiopia; 2 Centre for Crop and Disease Management, School of Molecular and Life Sciences, Curtin University, Perth, Western Australia, Australia; 3 Department of Statistics, College of Science, Bahir Dar University, Bahir Dar, Ethiopia; 4 Center for Environmental and Respiratory Health Research, Population Health, University of Oulu, Oulu, Finland; 5 Departemnt of Statistics, University of Pretoria, Pretoria, South Africa; 6 School of Health Systems and Public Health, Faculty of Health Sciences, University of Pretoria, Pretoria, South Africa; 7 Department of Midwifery, College of Health Sciences, Debre Tabor University, Debre Tabor, Ethiopia; 8 Department of Nursing, College of Health Sciences, Debre Tabor University, Debre Tabor, Ethiopia; 9 Department of Statistics, University of Johannesburg, Johannesburg, South Africa; 10 College of Health Solutions, Arizona State University, Tempe, Arizona, United States of America; Regional Health Care and Social Agency of Lodi, ITALY

## Abstract

**Background:**

Childhood immunization is an important part of public health efforts to reduce morbidity and mortality from diseases that can be prevented by vaccination. Sub-Saharan Africa (SSA) has the lowest childhood immunization coverage and the highest child mortality rate in the world. Therefore, this study aimed to assess community variation in childhood immunization and to identify determinant factors associated with childhood immunization using mixed effect count regression models.

**Method:**

This study used data from the 2012–2022 Demographic and Health Survey (DHS), which included 195,000 children between the ages of 12 and 23 months in 33 SSA countries. A various mixed-effects count regression model were employed to identify the variables associated with the prevalence of childhood vaccination.

**Result:**

In SSA, the mean average childhood immunization was 5.47 (95% CI = 5.46, 5.48), with an 8.38 variance. The mixed effect zero-inflated Poisson model fit the data the best, with the lowest DIC, AIC, and BIC values. The results of the model showed that working mothers, mothers with secondary or higher education (IRR = 1.119; 95% CI: 1.114, 1.125), rich wealth status (IRR = 1.077; 95% CI: 1.073, 1.082), having a health card (IRR = 1.265; 95% CI: 1.258, 1.271), being exposed to the media (IRR = 1.082; 95%CI: 1.077, 1.086), institutional delivery (IRR = 1.168; 95% CI: 1.163, 1.174), receiving eight or more ANC visits (IRR = 1.301; 95% CI: 1.288, 1.314), receiving vitamin A (IRR = 1.350; 95% CI: 1.344, 1.355), using a contraceptive (IRR = 1.187; 95% CI: 1.182, 1.192) and receiving Postnatal Care (PNC) (IRR = 1.075; 95% CI: 1.071, 1.080), were associated with a higher incidence of childhood immunization. While children in rural areas (IRR = 0.972; 95%CI: 0.968, 0.976) had a lower prevalence of childhood vaccinations than children in urban areas.

**Conclusion:**

SSA had a low coverage of childhood vaccination with significant disparities among countries. Therefore, it is essential to prioritize public health initiatives that target low-income households, rural mothers, uneducated parents, and those who have not utilized maternal health care services for the sake of increasing the coverage of childhood immunizations that in turn enhances the health of children. Furthermore, it is imperative to create policies and initiatives that tackle regional and national variations in childhood immunization rates and to actively work toward their implementation.

## Introduction

Child mortality is a significant global health problem, with Sub-Saharan Africa (SSA) still having the highest rates worldwide. The child mortality rate is estimated to be about 37 deaths per 1,000 live births, indicating that significant progress has been made in reducing child mortality worldwide [1,2]. However, over 55% of all child mortality worldwide occur in SSA. SSA has a child mortality rate of approximately 76 deaths per 1,000 live births in 2023, which is more than twice the global average and significantly higher than rates in other regions like Europe or North America, which are less than 10 deaths per 1,000 live births [2–4]. In SSA, infectious illnesses including malaria, pneumonia, and diarrhea combine with malnutrition continue to be the main causes of child mortality. Many countries in SSA are not on track to reach the Sustainable Development Goals (SDGs), a worldwide program that aims to reduce child mortality rate to at least 25 fatalities per 1,000 live births by 2030. Investments in healthcare facilities, immunizations, education, nutrition programs, and conflict resolution must continue if the area is to address its disproportionate burden of child deaths and narrow the gap with the rest of the world [1,5,6].

Immunization of children is crucial for reducing infant mortality and preventing millions of deaths annually [5–7]. About 84% of children worldwide receive essential immunizations, including those against polio, measles, diphtheria, tetanus, and pertussis (DTP3). Only 72% of children in Sub-Saharan Africa receive the third dose of DTP-containing vaccines, which is less than the global average. In 2023, 6.5 million children received only partial immunizations, while 14.5 million children globally received no vaccinations at all (referred to as zero-dose children) [7–10]. More than 60% of these 21 million children live in DR Congo, Ethiopia, India, Indonesia, Nigeria, Pakistan, Sudan, Yemen, Afghanistan, and Angola. Sub-Saharan Africa has high rates of child mortality from vaccine-preventable diseases as a result of poor immunization rates. One out of five children in Sub-Saharan Africa (SSA) lack access to necessary, life-saving vaccines. This leads to about 500,000 deaths every year and over 30 million vaccine-preventable deaths among African children under five [9–11].

Prior studies have been conducted in several SSA countries to identify the factors impacting children’s immunization [12–17]. Most of the studies were institutionally based, had few variables, and employed small-scale surveys [13,14]. Additionally, these studies examined the factors influencing childhood immunization using a binary logistic regression model [12–15,17]. While binary and ordinal logistic regression underestimates the total number of immunizations received by children, it does not provide enough information to examine the pattern of multiple immunizations because multiple immunizations are collapsed into a single unit to meet the requirements of binary and ordinal logistic regression [18,19]. Ordinal and binary logistic regression do not take over dispersion into account because they assume a constant variance. Additionally, it cannot take into consideration too many zeros in the data. This can lead to inaccurate inferences, biased parameter estimations, and loss of important information about the underlying numbers. As a result, our study addressed this problem by using a count regression model that is suitable for such count data. Therefore, this study used a count regression model appropriate for such count data in order to address the above-mentioned problem [20]. The Poisson regression model (PRM) is most commonly employed to analyze count data. The mean and variance of the count data are assumed to be equal in Poisson regression. This is referred to as the equi-dispersion assumption [21]. The fact is that many count datasets, including children vaccination records, frequently exhibit over dispersion, or a condition in which the variance exceeds the mean. Poisson regression can produce biased parameter estimates and underestimate standard errors when data is over dispersed, which can result in inaccurate statistical inference. The Negative Binomial model is more adaptable and more capable of managing real-world datasets with overdispersion or heterogeneity [22]. It prevents underestimating standard errors and offers more precise statistical inference by taking into account the additional variance in the data. Nevertheless, the Negative Binomial model is inappropriate in child immunization datasets because excess zeros may represent individuals who have not received any vaccinations because of barriers like limited access to healthcare or vaccine refusal. However, the NB model may not be sufficient due to the occurrence of excess zeros and over dispersion in the data [23]. In this instance, statistical analysis commonly uses zero-inflated and hurdle models to address excess zeros and overdispersion in count data [24–26]. These models are particularly useful for datasets that exhibit zero-inflation, where the proportion of zeros exceeds the expected value derived from a normal count distribution. Zero-inflated models are not suitable for under-dispersion, while hurdle models a flexible alternative that can capture both over- and under-dispersion [27].

Furthermore, the traditional count regression model was used in earlier studies to determine the factors affecting the number of immunization. The traditional count regression model assumes that each child is independent, ignoring the hierarchical nature of the data within communities. However, in this instance, the children are nested with the household and the region since traditional count regression models might not take into consideration the correlation between observations within the same group, which could result in estimates that are biased and misinterpretation [22]. This study used a multilevel count regression model to determine the factors affecting the number immunization in order to address this problem. These models extend traditional count models by introducing random effects to capture variability at different levels, including influences at the individual and group levels. This models also separate between-group and within-group variance to model these variations and produce more accurate estimates [28–30]. Therefore, this study aimed to assess childhood immunization variation among communities and identify determinant factors associated with childhood immunization using multilevel count regression models.

## Materials and methods

### Data sources and variables

The most recent Demographic and Health Surveys (DHS) data from 33 SSA countries between 2012 and 2022 were used in this study. Details on the DHS data can be found at: https://dhsprogram.com. To select the sample for each survey across the countries, DHS used two stage cluster sampling. Enumeration areas (EAs) are randomly chosen from the list of EAs for the first stage, and households in each EA are chosen at random for the second stage. Women between the ages of 15 and 49 are selected for an in-depth interview from the selected households [31]. To enable cross-country comparability, the datasets follow uniform protocols for questionnaire design, sampling, data collection, data cleaning, coding, and analysis. These DHS datasets were combined in order to assess the amount of childhood immunization and identify its determinants in SSA countries. In this study, 195868 children were included in the analysis using the KR dataset (Table 1).

**Table 1:** Pooled Demographic and Health Surveys (DHS) data from 33 Sub-Saharan countries, 2012–2022.

Country	Year of Survey	Weighted sample (n)	Weighted sample (%)
Ethiopia	2016	5337	2.7
Kenya	2022	10159	5.2
Angola	2017/18	7213	3.7
Burkina Faso	2021	6261	3.2
Benin	2017/18	6933	3.5
Burundi	2016/17	6767	3.5
Ghana	2022	5113	2.6
Cameroon	2018	4806	2.5
Gabon	2019/21	3258	1.7
Cote d’lvoire	2021	5414	2.8
Gambia	2019/20	4391	2.2
Guinea	2018	3910	2.0
Liberia	2019/20	2949	1.5
Lesotho	2014/15	2460	1.3
Madagascar	2021	6532	3.3
Mali	2018	4947	2.5
Mauritania	2019/21	5842	3.0
Malawi	2015/16	9257	4.7
Niger	2012	7089	3.6
Mozambique	2022	4956	2.5
Nigeria	2018	16291	8.3
Togo	2013/14	4815	2.5
Chad	2014/15	10264	5.2
Congo	2011/12	6219	3.2
Congo DR	2013/14	10606	5.4
Comoros	2012	1947	1.0
Namibia	2013	3803	1.9
Uganda	2016	7710	3.9
Rwanda	2019	4363	2.2
Tanzania	2022	5679	2.9
Zambia	2018	5359	2.7
Zimbabwe	2015	3300	1.7
South Africa	2016	1918	1.0
Total		195868	100.0

### Study variables

#### Dependent variable.

The dependent variable in this study is the number of immunizations the children have received. The mothers disclosed whether or not their children had received a particular immunization. The data on each of the eight vaccines were then combined to determine the total number of immunizations a child has received. According to the WHO definition [32–34], the eight different types of vaccines are three doses of DPT, three doses of polio, one dose of measles, and one dose of BCG.

#### Independent variables.

Both community-level and individual-level factors were identified as potential predictors to childhood immunization. These variables have been selected based on the previous literature [12–17]. The individual-level variables include the age of mothers (15–24, 25–34 and 35–49 years), wealth index (poor, middle, rich), mothers employment status (housewives, working any sector), maternal education level (no education, primary education and secondary and above), father education level (no education, primary education and secondary and above), husband’s occupation (not working, working), birth order (1, 2–4, 5 and above), Number of living children (1, 2–3, 4 and above), women’s decision-making capacity (women alone, women and her husband, husbands alone), contraceptive use (no, yes), ANC visits during pregnancy, place of delivery, sex of household head (male, female), marital status (never married, married, divorced/widowed) and PNC visit (no, yes),. Moreover, residence (rural, urban), distance to a health facility (big problem, not a big problem), exposure to mass media (no, yes), region, and country and SSA region (eastern region of Africa, central region of Africa, southern region of Africa, western region of Africa) were considered as a community-level variable.

### Statistical software

The data was extracted, cleaned, and coded using the Statistical Software for Social Sciences (SPSS) version 25. After data management the data was exported to R software version 4.4.3 and analyzed with the glmmTMB package.

### Statistical analysis

In epidemiology and public health research, count data are commonly used to represent the number of particular outcomes or events occur in a given community. These data types are often non-negative integers that can describe a variety of occurrences, such as the number of immunizations received by a child, the number of disease instances, hospital admissions, or deaths within a specific time period or geographic area. The count regression model is the most adaptable regression model for assessing count response data without dichotomization [35]. A multistage stratified cluster sampling technique was used to gather DHS data since children were nested with households, which were nested with regions. This multistage stratified cluster sampling technique frequently introduces multilevel dependency or correlation between data, which could have an impact on model parameter estimations. The interdependence of observations in multistage stratified cluster samples is frequently caused by multiple stages of the hierarchy. The traditional count regression model’s application is inappropriate and reasonable since it assumes that there is no relationship between individual data [30]. In order to solve the issue of dependencies between individual observations in DHS data, this study employed six multilevel count regression models, including Poisson, Negative Binomial, Zero-Inflated Poisson, Zero-Inflated Negative Binomial, Hurdle Poisson, and Hurdle Negative Binomial.

Poisson regression is the most popular and fundamental model that specifically takes into account the nonnegative integer-valued component of the count outcome variable. The basic assumption of the Poisson regression are the variance and mean of the dependent variable are assumed to be equal [21]. The inherent correlation between observations is taken into consideration by using a multilevel Poisson regression model with random effects. The two-level Poisson regression model for a count $y_{ij}$ for $i^{th}$ individual women in $j^{th}$ region can be written as: yijλij poisson(mij,λij)

The assumption that the mean and variance of the data are equal in Poisson regression may not hold true in real-world datasets, especially ones with a high degree of variation or a significant number of extreme values. Poisson regression may produce biased estimates and underestimated standard errors in this case. Negative Binomial (NB) regression is superior to Poisson regression in this situation when handling over-dispersed count data, when the variance is greater than the mean. The NB model is more robust for over-dispersed data since it incorporates an extra dispersion parameter that gives more modeling flexibility. This flexibility leads to better fit and more accurate parameter estimates when the Poisson assumption is violated. However, the traditional negative binomial model is insufficient when there is dependence between observations since it assumes that observations are independent. The multilevel negative binomial regression model accounts for the dependence between data within clusters or groups and generates more accurate estimates of the correlations between variables by appropriately reflecting the variance at several levels [22]. The two level negative binomial regression models are given by:


$$\begin{array}{c} P\left(y_{ij},\mu ,\alpha \right)=\frac{\Gamma \left(y_{ij}+\frac{\text{1}}{\alpha }\right)}{y_{ij}!\Gamma \left(\frac{\text{1}}{\alpha }\right)}{\left(\text{1}+\alpha \mu \right)}^{-\frac{\text{1}}{\alpha }}{\left(\text{1}+\frac{\text{1}}{\alpha \mu }\right)}^{-y_{ji}}\;y_{ij}\geq \text{0}\text{ and }\alpha >\text{0} \end{array}$$

Where α is the over dispersion parameter and Γ(.) is the gamma function when α=0 the NB distribution is the same as Poisson distribution. The mean and variance are expressed as:


$$\begin{array}{c} E\left(y_{i}\right)=\mu_{i}=\text{exp}\left(x_{i}{\;}^{T}\beta \right),\;Var(y_{i})=\mu_{i}(\text{1}+\alpha \mu_{i}) \end{array}$$

The standard link function for the two level Poisson and NB model is obtained by incorporating region-specific random effects in the standard Poisson/NB model, and we get


$$\begin{array}{c} log\left(\mu_{ij}\right)={x_{ij}}^{T}\beta +u_{j} \end{array}$$

Where $X_{ij}$ represent the first and the second level explanatory variable, β are (p+1)×1 vector of regression coefficients and $u_{j}$ stand for the random intercepts at region level and are assumed to follow a normal distribution with constant variance.

For over-dispersed counts, the negative binomial regression model assumes that the count data follows a specific distribution. However, it may not be suitable for situations in which the number of zeros is excessive because of distinct underlying processes. Zero-inflated models are used in studies that have an excess of zeros in the data, which is common in count data settings such as the study of childhood immunization rates. The extra number of children who have not had any vaccinations can be taken into account in this situation by using a zero-inflated model [26]. Zero-inflated models that integrate count models and logistic regression. The Poisson or negative binomial model is used to model positive counts, while the logistic model separates zeros from positive counts. There are two common models for zero-inflated count data: zero-inflated Poisson (ZIP) and zero-inflated negative binomial (ZINB). The ZIP model [24] is a dual-state technique for modeling data with a significant number of zeroes or more zeroes than one would expect in a traditional Poisson or negative binomial model, whereas the ZINB model is more flexible and can handle over-dispersion caused by both unobserved heterogeneity and excess zeroes [36]. Assuming statistical independence between individual observations, the standard ZIP regression can be used to model count data that contains excess zeros. However, simultaneous correlation and zero-inflation might occur due to the hierarchical study design, which violating the assumption that individual observations are statistically independent [37]. To handle correlated count data with extra zeros, a multi-level ZIP regression model was developed. The two level ZIP models’ probability distribution is shown as follows:


$$\begin{array}{c} p(Y_{ij}=y_{j})=\left\{ \begin{array}{c} \pi_{ij}+(1-\pi_{ij})exp(-\mu )\;,\;\;\;\;\;\;\;\;\;\;\;\;\;\;if\;y_{ij}=0\\ (1-\pi_{ij})\frac{exp(-\mu )\mu^{y_{ij}}}{y_{ij}!},\;\;\;\;\;\;\;\;\;\;\;\;\;\;\;if\;y_{{\;}_{i}j}=1,2,...... \end{array}\right.\;\;\;\;\;\;\;\;\;\;0\leq \pi_{ij}\leq 1 \end{array}$$

where $Y_{ij}$ indicates the number of immunization the $i^{th}$ children in the $j^{th}$ region, and μ is the mean for the Poisson distribution. When dealing with overdispersion and excess zeros in count data, ZINB regression is better than ZIP [38]. We use a multilevel ZINB regression with random effects to account for excess zero, over-dispersion, and data dependency problems [39]. Let $Y_{ij}(i=1,2,.....,n;$j=1,2,.....,n_j be a count say, the number of immunizations the $i^{th}$ children in$j^{th}$ the region follows a ZINB distribution:


p(Yij=yij)={πij+(1−πij)(1+αμ)−1α,ifyij=01−πijΓ(yij+1α)yij!Γ(1α)(1+αμij)−1α(1+1αμ)−yij,ifyij>00≤πij≤1

With parameters μ≥0 for the mean and α>0 over-dispersion

The vector representation of the two-level ZINB and ZIP regression model is as follows:


$$\begin{array}{c} \eta_{ij}=log\left(\mu_{ij}\right)={x_{ij}}^{T}\beta +u_{j} \end{array}$$

$$\begin{array}{c} \xi_{ij}=log\left(\frac{\pi_{ij}}{1-\pi_{ij}}\right)={z_{ij}}^{T}\gamma +w_{j} \end{array}$$

The explanatory variable $X_{ij}$ and $Z_{ij}$ appearing in the respective negative binomial and logistic components are not necessarily the same β and γ are the corresponding vectors of regression coefficients [39,40]. The vectors $u_{j}$ and $w_{j}$ denote the enumeration area-specific random effects for simplicity of presentation. The random effect u and w assumed to be independent and normally distributed with mean zero and variance ${\sigma_{u}}^{\text{2}}$ and ${\sigma_{w}}^{\text{2}}$ respectively.

Hurdle models, in contrast to ZI models, are two-component mixture models that have a zero mass and a positive observations component derived from a truncated count distribution, such as a truncated NB or Poisson distribution [25]. The hurdle model is flexible and can handle both under- and over-dispersion data, whereas ZI models are exclusively used to analyze over-dispersion data. [41]. While hurdle models can manage zero-deflation, ZI models cannot handle it at any level of a factor, leading to parameter estimates near infinity for the logistic component [25]. The Poisson Hurdle model with two levels is expressed as follows:


$$\begin{array}{c} p(Y_{ij}=y_{ij})=\left\{ \begin{array}{c} \pi_{ij}\;\;\;\;\;\;\;\;\;\;\;\;\;\;\;\;\;\;\;\;\;\;\;\;\;\;\;\;\;\;\;\;\;\;\;\;\;\;\;\;\;\;\;\;\;\;\;\;\;\;\;\;\;\;\;\;\;\;\;\;\;\;\;\;\;if\;y_{ij}=0\\ (1-\pi_{ij})\frac{exp(-\mu )\mu^{y_{ij}}}{(1-exp(-\mu )y_{ij}!}\;\;\;\;\;\;\;\;\;\;\;\;\;\;\;\;\;if\;y_{ij}=1,2,..... \end{array}\right.\;\;\;\;\;\;\;\;\;0\leq \pi_{ij}\leq 1 \end{array}$$

where $Y_{ij}$ is the number of ANC visit for the $i^{th}$ women in the $j^{th}$ region $\mu_{ij}$ is the mean and $\pi_{ij}$ is zero proportion parameters. The multilevel HNB model is the most widely used method for distributing the non-zero count component to allow for over-dispersion. The two level HNB model is given as:


p(Yij=yij)={πij,ifyij=0(1−πij)Γ(yij+1α)yij!Γ(1α)(1+αμij)−1α(1+1αμ)−yij,ifyij>00≤πij≤1

with parameters μ≥0 for the mean and α>0 for over-dispersion

In the regression setting, both the mean $\mu_{ij}$ and zero proportion $\pi_{ij}$ parameters are related to the vectors of known explanatory variables $x_{ij}$ and $z_{ij}$ respectively. Moreover, responses within the same region are likely to be correlated. To accommodate the inherent correlation, random effects $u_{j}$ and $w_{j}$ are incorporated in the linear predictors $\eta_{ij}$ for the Poisson part and $\xi_{ij}$ for the zero part. The two levels Poisson Hurdle and Negative binomial Poisson mixed regression model is


$$\begin{array}{c} \eta_{ij}=log\left(\mu_{ij}\right)={x_{ij}}^{T}\beta +u_{j} \end{array}$$

$$\begin{array}{c} \xi_{ij}=log\left(\frac{\pi_{ij}}{1-\pi_{ij}}\right)={z_{ij}}^{T}\gamma +w_{j} \end{array}$$

**Fig 1:**
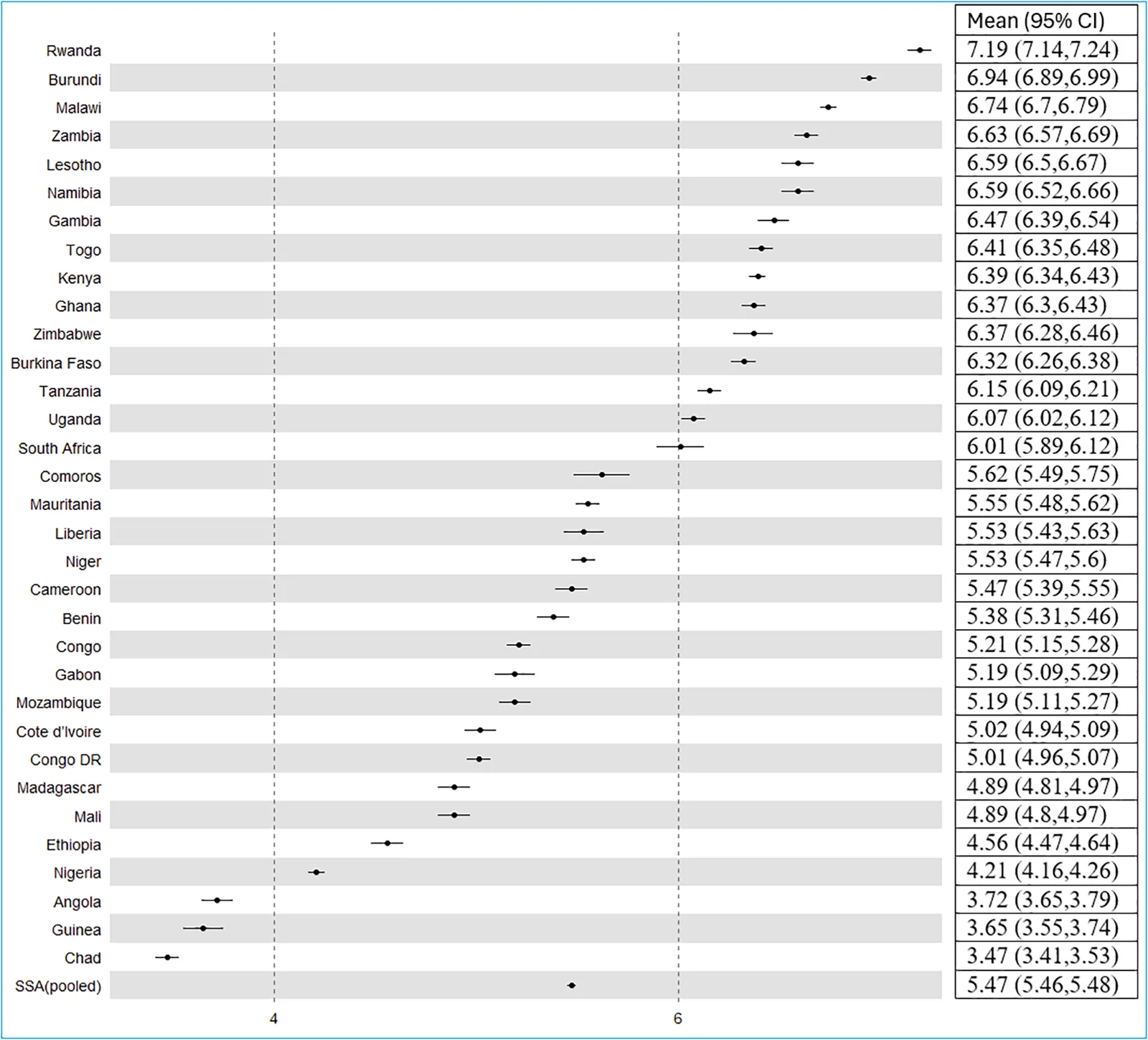
Forest plot of the mean number of vaccines taken by the children across SSA countries.

Where β and γ are the corresponding (p+1)×1 and (q+1)×1 vector of regression coefficients. The random effects $u_{j}$ and $w_{j}$ are assumed to be independent and normally distributed with mean 0 and variance ${\sigma_{u}}^{\text{2}}$ and ${\sigma_{w}}^{\text{2}}$, respectively (Fig 1) [42].

Deviance information criteria (DIC), Akaike’s information criterion (AIC), and Bayesian information criteria (BIC) were used to compare the models. The model that best fits the data is the one with the lowest Bayesian Information Criterion (BIC), Akaike’s Information Criterion (AIC), and Deviance Information Criteria (DIC). All variables were categorized as statistically significant if their P-values were less than or equal to 0.05. The fixed effects of the logistic and count parts were reported using the adjusted odds ratio (AOR) with a 95% CI and the incidence rate ratio (IRR) with a 95% CI, respectively. Random effect was measured using Intra-class Correlation Coefficient (ICC) and Variance. The ICC was determined using the following formula and represents the variation in immunization rates among regions:


$$\begin{array}{c} ICC=\frac{V_{A}}{V_{A}+\;3.29} \end{array}$$

Where $V_{A}$ is the estimated variance in each model [43].

## Result

The analysis included 195, 868 children between the ages of 12 and 23 months. About 72,523 (37.0%) of children have received all eight types of immunizations, 172,638 (88.1%) have received at least one vaccine, and 23230 (11.9%) have not received any vaccines. The average number of immunizations was 5.47 with a variance of 8.38. The variance-to-mean ratio is 1.53, which suggests that there is over-dispersion in the data (Table 2).

**Table 2:** The number of vaccines taken by the children.

Number of vaccines	Frequency	Percent
0	23230	11.9
1	10805	5.5
2	5951	3.0
3	13488	6.9
4	7576	3.9
5	14177	7.2
6	9701	5.0
7	38417	19.6
8	72523	37.0
**Total**	**195868**	**100.0**
**Mean**	**5.47**	
**Variance**	**8.38**	

### The magnitude of childhood immunization across SSA countries

The average number of childhood immunization in SSA countries was presented in Fig 1. The average number of childhood immunization in SSA was 5.47 (95% CI = 5.46, 5.48). The average number of childhood immunizations in each of the SSA countries varied significantly. Rwanda (7.19: 95%CI = 7.14, 7.24), Burundi (6.94: 95%CI = 6.89, 6.99), and Malawi (6.74: 95%CI = 6.70, 6.79) have the highest average number of childhood immunizations. Chad (3.47: 95%CI = 3.41, 3.53), Guinea (2.62: 95%CI = 3.55, 3.74), and Angola (3.72: 95%CI = 3.65, 3.79) have the lowest average number of childhood immunizations (Fig 1).

### Specific immunization coverage in Sub-Saharan Africa region

**Fig 2:**
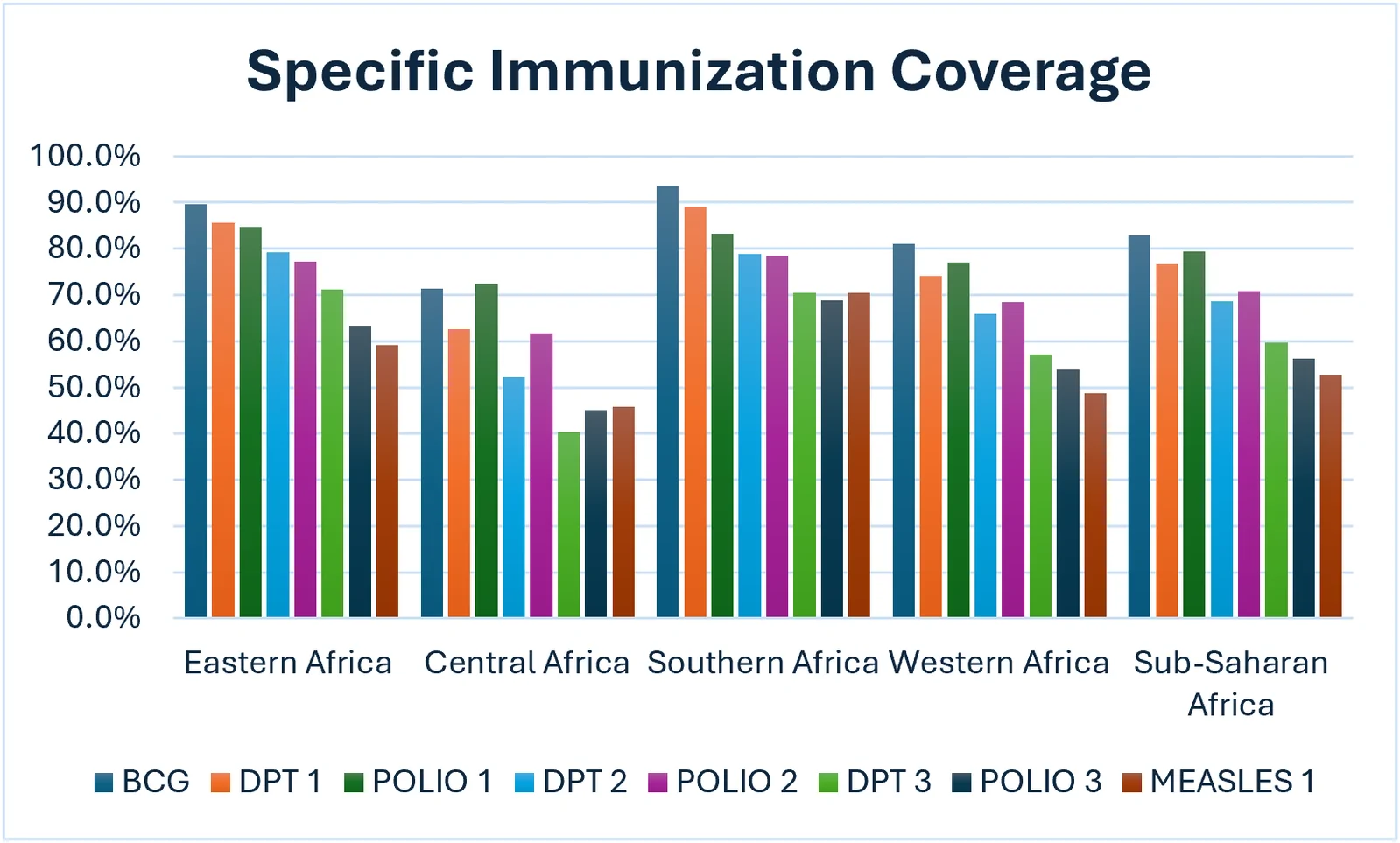
Specific immunization coverage in sub-Saharan Africa region.

Specific immunization coverage in SSA region were summarized in Fig 2. The coverage of BCG immunization in SSA countries was higher (82.8%) and the coverage of MEASLES immunization was lower (52.8%). The coverage of all immunization in Southern and Easter Africa were higher and lower in Central Africa (Fig 2).

### Socio-demographic characteristics of study participants

The mean number of childhood immunizations based on the socio-demographic variables of studies participants is shown in Table 3. About 36.0% of the mothers had no formal education, and 45.4% of the mothers were between the ages of 25–34 years. The majority, (58.1%) of mothers were employed, and over half (68.1%) of them lived in rural areas. About 45.4% of the fathers had no formal schooling and more than half of the fathers had employed. About 13.1% of pregnant mothers did not receive Antenatal Care (ANC) visits and the majority, (69.9%) of children were born in health institution. One-third, (33.1%) of mothers who had Postnatal Care (PNC) checkups and about 46.6% of children were from poor household (Table 3).

**Table 3:** Mean number of immunizations by socio-demographic characteristics.

Variable	Frequency	Percentage	Mean number of immunizations (95% CI)
**Age of mother**			
15-24	64598	33.0	5.26(5.24, 5.28)
25-34	88994	45.4	5.55(5.53, 5.57)
35-49	42276	21.6	5.63(5.60, 5.66)
**Mother education level**			
No education	70550	36.0	4.61(4.59, 4.64)
Primary education	63965	32.7	5.78(5.76, 5.80)
Secondary and above	61353	31.3	6.13(6.12, 6.15)
**Mothers Occupation**			
Housewives	82040	41.9	5.14(5.12, 5.16)
Working any sector	113828	58.1	5.70(5.69, 5.72)
**Father education level**			
No education	88921	45.4	4.99(4.97, 5.01)
Primary education	44964	23.0	5.80(5.77, 5.83)
Secondary and above	61983	31.6	5.92(5.90, 5.94
**Father occupation**			
Not working	86582	44.2	5.61(5.59, 5.63)
Working	109286	55.8	5.36(5.34, 5.38)
**Wealth index**			
Poor	91178	46.6	5.03(5.01, 5.05)
Middle	38792	19.8	5.55(5.53, 5.58)
Rich	65898	33.6	6.03(6.01, 6.05)
**Family size**			
1-3	26282	13.4	5.75(5.72, 5.79)
4-6	86279	44.0	5.57(5.56, 5.59)
7 and above	83307	42.5	5.27(5.25, 5.29)
**Number of living children**			
1	45754	23.4	5.72(5.69, 5.74)
2-3	73968	37.8	5.54(5.52, 5.56)
4 and above	76146	38.9	5.26(5.24, 5.28)
**Birth order**			
First	42590	21.7	5.74(5.72, 5.78)
2-4	94434	48.2	5.54(5.52, 5.56)
5 and above	58844	30.0	5.26(5.24, 5.28)
**Marital status**			
Never married	59431	30.3	5.55(5.53, 5.78)
Married	130951	66.9	5.42(5.40, 5.44)
Divorced/widowed	5486	2.8	5.81(5,74, 5.88)
**Sex of child**			
Male	99099	50.6	5.47(5.45, 5.49)
Female	96769	49.4	5.47(5.45, 5.49)
**ANC visit**			
No visit	25575	13.1	2.96(2.92, 3.00)
1-3	61310	31.3	5.47(5.45, 5.49)
4-7	96902	49.5	6.05(6.03, 6.06)
8 and above	12081	6.2	6.16(6.11, 6.20)
**Place of delivery**			
Home	58895	30.1	4.02(4.00, 4.05)
Health center	136973	69.9	6.09(6.08, 6.11)
**Received Vitamin A1**			
No	89678	45.8	4.04(4.02, 4.06)
Yes	106190	54.2	6.68(6.67, 6.70)
**Has health card**			
No	54579	27.9	3.29(3.27, 3.32)
Yes	141289	72.1	6.31(5.10, 5.13)
**PNC**			
No	131103	66.9	5.12(5.10, 5.13)
Yes	64765	33.1	6.18(6.16, 6.20)
**Size of child at birth**			
Average	101348	51.7	5.55(5.53, 5.57)
Smaller than average	30750	15.7	5.16(5.13, 5.19)
Larger than average	63770	32.6	5.50(5.48, 5.52)
**Type of birth**			
Single birth	192642	98.4	5.47(5.46, 5.48)
Multiple birth	3226	1.6	5.65(5.55, 5.74)
**Sex of household head**			
Male	153069	78.1	5.43(5.41, 5.44)
Female	42799	21.9	5.63(5.60, 5.65)
**Residence**			
Urban	62438	31.9	5.86(5.84, 5.88)
Rural	133430	68.1	5.29(5.27, 5.31)
**Distance to a health facility**			
Big problem	86840	44.3	5.08(5.06, 5.10)
Not a big problem	109028	55.7	5.78(5.76, 5.80)
**Contraceptive use**			
No	133847	68.3	4.92(4.90, 4.94)
Yes	62021	31.7	6.66(6.65, 6.68)
**Mass media exposure**			
Not exposed	75832	38.7	4.79(4.77, 4.82)
Exposed	120036	61.3	5.90(5.88, 5.91)
**Tetanus injections before birth**			
Not received	50735	25.9	4.30(4.27, 4.33)
1-2	113823	58.1	5.83(5.82, 5.85)
3 and above	31310	16.0	6.06(6.03, 6.09)

Additionally, the lowest mean number of childhood immunizations (5.26) was found among mothers aged 15–24. The mean number of childhood immunizations was lowest among uneducated mothers (4.61), whereas the mean number of childhood immunizations was highest among those with higher education levels (6.13). Fathers with secondary education and above had the highest mean number of childhood immunizations (5.92), whereas fathers with no education had the lowest mean number of childhood immunizations (4.99). The mean number of childhood immunizations (6.09) was higher among children born at health institutions. Children with health cards had a greater mean number of childhood immunizations (6.31). The mean number of childhood immunizations was higher (6.18) among mothers who had PNC checkups. The mean number of childhood immunizations was higher among mothers who received three or more tetanus injections before to birth (6.06). Mothers who used contraceptives had a greater mean number of childhood vaccinations (6.66) (Table 3).

### Model selection criteria

The results of the model selection criteria are summarized in Table 4. The zero-augmented (zero-inflated and hurdle) models can provide better fits due to excessive zero values. The Poisson distribution performs better than the NB distribution in multilevel zero-inflated and hurdle models. As a result, the multilevel HP model fits data better than other count regression models due to its lower deviance, AIC, and BIC values (Table 4).

**Table 4:** Model selection criteria for the multilevel count regression models.

Model	Deviance	AIC	BIC
**MP**	916315.8	916397.8	916815.4
**MNB**	916771.6	916855.6	917283.4
**MZIP**	**835315.7**	**835479.7**	**836314.8**
**MZINB**	835625.9	835791.9	836637.3
**MHP**	835368.9	835532.9	836368.1
**MHNB**	835654.3	835820.3	836665.7

### Determinants of childhood immunization in Sub-Saharan Africa

The results of the multilevel zero inflated Poisson models are shown in Table 5. The fixed and random effects of the Poisson and Bernoulli components were provided by this model. The Poisson component displays the levels of childhood immunizations or the Incidence of Relative Risk (IRR). Women aged 35–49 years had an expected number of childhood immunizations 1.095 (IRR = 1.095; 95%CI: 1.086, 1.104) times greater than those aged 15–19 years. The expected number of childhood immunizations was 1.119 (IRR = 1.119; 95% CI: 1.114, 1.125) times greater for mothers with secondary or higher education than for those uneducated mothers. Compared to fathers who were uneducated, fathers who had secondary or higher education had 1.064 (IRR = 1.064; 95%CI: 1.059, 1.068) times the expected number of childhood immunizations. Compared to urban areas, the expected number of children immunizations in rural areas was 0.972 (IRR = 0.972; 95%CI: 0.968, 0.976) times lower. The incidence of childhood immunization was 1.077 (IRR = 1.077; 95% CI: 1.073, 1.082) times higher in rich households than in poor households.

**Table 5:** Determinants of childhood immunization in Sub-Saharan Africa.

Variable	Poisson part	Bernoulli part
	IRR (95%CI)	AOR (95%CI)
**Residence**		
Urban	1	1
Rural	0.972(0.968, 0.976)*	2.069(1.999, 2.142)*
**Mother education level**		
No education	1	1
Primary education	1.092 (1.087, 1.100)*	0.875 (0.829, 0.923)*
Secondary and above	1.119 (1.114, 1.125)*	0.784 (0.732, 0.840)*
**Wealth index**		
Poor	1	1
Middle	1.034 (1.030, 1.041)*	0.916 (0.869, 0.965)*
Rich	1.077 (1.073, 1.082)*	0.860 (0.809, 0.915)*
**Father education level**		
No education	1	1
Primary education	1.067 (1.062, 1.073)*	0.504(0.486, 0.523)*
Secondary and above	1.064 (1.059, 1.068)*	0.372(0.358, 0.385)*
**Mother occupation**		
Housewives	1	1
Employed	1.053 (1.049, 1.057)*	0.644(0.626, 0.662)*
**ANC visit**		
No visit	1	1
1-3	1.221 (1.211, 1.231)*	0.750 (0.707, 0.796)*
4-7	1.287 (1.278, 1,297)*	0.621 (0.583, 0.662)*
8 and above	1.301 (1.288, 1.314)*	0.612 (0.544, 0.688)*
**PNC**		
No	1	1
Yes	1.075 (1.071, 1.080)*	0.561 (0.531, 0.592)*
**Place of delivery**		
Home	1	1
Health facility	1.168 (1.163, 1.174)*	0.147(0.143, 0.152)*
**Age of women**		
15-24	1	1
25-34	1.049 (1.042, 1.055)*	0.778 (0.735, 0.824)*
35-49	1.095 (1.086, 1.104)*	0.634 (0.584, 0.688)*
**Family size**		
1-3	1	1
4-6	0.981 (0.975, 0.987)*	1.110 (1.040, 1.184)*
7 and above	0.974 (0.968, 0.981)*	1.165 (1.088, 1.247)*
**Contraceptive use**		
No	1	1
Yes	1.187 (1.182, 1.192)*	0.547 (0.515, 0.581)*
**Distance to a health facility**		
Big problem	1	1
Not a big problem	1.051 (1.047, 1.055)*	0.515(0.500, 0.529)*
**Mass media exposure**		
Not exposed	1	1
Exposed	1.082 (1.077, 1.086)*	0.348(0.339, 0.359)*
**Previous birth interval**		
Less than 24 month	1	1
24 month or above	1.038 (1.032, 1.044)*	0.690(0.665, 0.717)*
**Received Vitamin A1**		
No	1	1
Yes	1.350 (1.344, 1.355)*	0.132(0.127, 0.137)*
**Has health card**		
No	1	1
Yes	1.265 (1.258, 1.271)*	0.067 (0.063, 0.070)*
**Tetanus injections before birth**		
Not received	1	1
1-2	1.09(1.08, 1.10)*	0.689 (0.655, 0.725)*
3 and above	1.12 (1.11, 1.13)*	0.590 (0.549, 0.633)*
**SSA region**		
Eastern region	1	1
Central region	1.553 (1.232, 1.957)*	0.891 (0.872, 0.910)*
Southern region	2.830 (1.791, 4.470)*	0.925 (0.890, 0.962)*
Western region	2.019 (1.623, 2.511)*	0.934 (0.917, 0.953)*
**Random effect**		
Between-region Area variance $\left({\sigma_{u}}^{\text{2}}\right)$	1.01(0.98, 1.05)*	0.42(0.39, 0.45)*
ICC	23.49%	11.32%

1 = reference category of the categorical variable.

*Significant at 5% level of significance.

The incidence of childhood immunization was 1.082 (IRR = 1.082; 95%CI: 1.077, 1.086) times greater for women who used mass media than women who did not use mass media. The incidence of childhood immunizations was 1.051 (IRR = 1.051; 95% CI: 1.047, 1.055) times higher among mothers whose distance to a health facility is not a big problem than among mothers whose distance is a big problem. The incidence of childhood immunization was 1.187 (IRR = 1.187; 95% CI: 1.182, 1.192) times greater for women who used contraception than for those who did not used contraception. The incidence of childhood immunizations was 1.301 (IRR = 1.301; 95% CI: 1.288, 1.314) times higher for mothers who received eight or more ANC visits during pregnancy than mothers who did not received ANC visits during pregnancy. The incidence of childhood immunizations was 1.168 (IRR = 1.168; 95% CI: 1.163, 1.174) times higher for children born in a health facility than for those delivered at home. The incidence of childhood immunization was higher in Central Africa (IRR = 1.129; 95% CI: 1.121, 1.137), Western Africa (IRR = 1.260; 95% CI: 1.244, 1.276), and Southern Africa (IRR = 1.285; 95% CI: 1.278, 1.292) than in Eastern Africa. The ICC found that differences in childhood immunization rates were 23.49% between regions (Table 5).

The childhood immunization is significantly correlated with the following factors in the zero-inflated Poisson model: the woman’s education, occupation, husband’s occupation, media exposure, distance to health facilities, obtaining the money needed for treatment, residence, age at first birth, use of contraceptives, women’s decision-making ability, pregnancy desired, birth order, health insurance, pregnancy complication, wealth index, and region. Mothers with secondary or higher education were 0.784 (AOR = 0.784, 95% CI: 0.732, 0.840) times less likely to have zero childhood immunizations than those without education. The odds of a mother not receiving childhood immunization were 0.644 (AOR = 0.644; 95% CI, 0.626, 0.662) times lower for working mothers than for housewives. Primary school-educated fathers were 0.504 (AOR = 0.504, 95% CI: 0.486, 0.523) times less likely to have zero childhood immunizations than fathers without any formal education. Rich mothers had a 14.00% (AOR = 0.860, 95% CI: 0. 809, 0.915) lower chance of having no childhood immunizations than poor mothers. Compared to urban children, rural children had 2.069 (AOR = 2.069; 95% CI: 1.999, 2.142) times higher odds of having no childhood immunizations. Compared to mothers who did not use contraception, mothers who used contraceptives had 0.547 (AOR = 0.547; 95%CI: 0.515, 0.581) times reduced odds of having zero childhood immunizations. Mothers who used mass media had 0.348 (AOR = 0.348; 95%CI: 0.339, 0.359) times lower odds of having zero childhood immunizations than mothers who did not use any mass media. Compared to the child without a vaccination card, the child with a vaccination card had an estimated 0.067 (AOR = 0.067; 95%CI: 0.063, 0.070) times lower odds of not obtaining any childhood immunizations. The odds of having no childhood immunization were 0.147 (AOR = 0.147; 95%CI: 0.143, 0.152) times lower for children born in a health facility than for those born at home. Mothers who receive checkup postnatal care have a 0.561 (AOR = 0.561; 95%CI: 0.531, 0.592) lower chance of having zero childhood immunizations than mothers who do not receive checkup postnatal care. Mothers who had three or above tetanus injections during pregnancy had 0.590 (AOR = 0.590; 95%CI: 0.549, 0.633) times lower odds of having zero childhood immunizations than mothers who did not receive a tetanus injections during pregnancy.

Mothers who received eight or more ANC visits during pregnancy had a 0.612 (AOR = 0.612; 95%CI: 0.544, 0.688) lower chance of having zero childhood immunizations than mothers who did not receive ANC visits during pregnancy. The variance components for the random effects is 0.42 (95% CI: 0.39, 0.45) show that there are significant geographical disparities in the non-childhood immunizations. According to the ICC results, the regional difference in the non-childhood immunizations was 11.32% (Table 5).

## Discussion

Childhood immunization is essential for reducing childhood morbidity and mortality as well as preventing infectious illnesses. The mean number of childhood immunization in SSA was 5.47 (95% CI = 5.46, 5.48). The study found that the mixed effect zero-inflated Poisson model had the minimum DIC, AIC, and BIC statistic values among the other mixed effect count models, making it the best model for identifying the factors influencing the number of childhood immunization. Similar findings from earlier research indicated that the mixed effect zero-inflated Poisson model was the best model for determining the factors that contribute to the incidence of childhood immunization [44]. The mixed effect zero-inflated Poisson regression model revealed that maternal education, occupation, age of mother, father education, media exposure, distance to health facilities, number of ANC visits, PNC, residence, place of delivery, received vitamin A, contraceptive use, family size, wealth index, tetanus injections before birth, presence of health card, previous birth interval and SSA region were found to be significantly correlated with the incidence of childhood vaccination in SSA countries.

The study also found that maternal age was a significant predictor of the incidence of childhood immunization. This is in line with the studies carried out in Ethiopia [14,45], Somalia [46], Nigeria [15,47], sub-Saharan Africa [12] and Bangladesh [48]. The incidence of childhood immunization was higher in older mothers than in younger mothers. This is due to the fact that older women have more experience with the significance of immunization, are more conscious of the demands of healthcare, have more financial security, have easier access to medical services, and feel more pressure to follow immunization schedules. Compared to housewife mothers, working mothers had a greater incidence of childhood immunization. This result is consistent with studies conducted in in Nigeria [15,47], sub-Saharan Africa [12], Bangladesh [48] and Sumatera, Indonesia [49]. The reason for this is that working mothers tend to have more access to healthcare facilities, higher levels of education and immunization awareness, better financial stability, better social networks and health insurance benefits from their professions.

The incidence of childhood immunization was higher among educated parents than uneducated parents. This finding was in agreement with a study done in Ethiopia [14], Somali Region, Eastern Ethiopia [13], Somalia [46], Nigeria [15,47], three West African countries [16] and Sumatera, Indonesia [49]. The possible explanation is that parents with higher levels of education are more likely to recognize the value of immunizations, follow vaccination schedules, and actively seek out immunization programs. Educated mothers also have better access to health information and are more inclined to advocate for their children’s health.

Children in urban regions received more vaccinations than those in rural areas. This finding is in line with studies done in Ethiopia [14,17], Somali Region, Eastern Ethiopia [13], Somalia [46], sub-Saharan Africa [12], and Sumatera, Indonesia [21]. The explanation for this might be that urban regions tend to have higher immunization rates since they have better access to healthcare facilities, better infrastructure, and more vaccines available. In contrast, there are a number of obstacles to immunization in rural areas, including transportation problems, limited number of health experts to administer vaccines, and long travel times to medical facilities.

Rich households had a greater childhood immunization rate than poor households. This finding is consistent with a study done in Ethiopia [17], Somali Region, Eastern Ethiopia [13], Somalia [46], Nigeria [15], sub-Saharan Africa [12], and Bangladesh [48]. This could be due to the fact that rich households are more likely to have access to immunization services in the form of paid time off from work, vaccine purchases, or transportation to health facility. Additionally, wealthy households are more likely to have access to the media, the internet, and health specialists who can explain the value of immunizations, which is frequently correlated with better information access.

Mothers who had ANC visits during pregnancy had a higher incidence of childhood vaccinations than mothers who did not receive ANC visits during pregnancy. This finding is supported by a study done in Ethiopia [17], Nigeria [15,47], Northern Nigeria [50], three West African countries [16] and sub-Saharan Africa [12]. The potential explanation could be that mothers who regularly attend ANC visits are more likely to be aware of immunization schedules and frequently receive encouragement from health professionals to vaccinate their children.

Mothers who used mass media had a higher incidence of childhood immunization than mothers who did not used mass media. The finding is agreed with previous researches in Ethiopia [17], Nigeria [47], Bangladesh [48,51], three West African countries [16] and sub-Saharan Africa [12]. The possible explanation for this could be that parents who are exposed to the media are more likely to recognize the value of immunizing their children, overcome misconceptions, reduce vaccine hesitancy, and be more driven to adhere to immunization schedules. Furthermore, parents may be reminded of immunization possibilities by media exposure, which could help to reduced missed doses.

The prevalence of childhood immunization was higher among women who used contraceptives than among those who did not used contraceptives. This is consistent with the studies conducted in Kenya and Uganda [52] and Nepal, Senegal, and Zambia [14]. This may be explained by the fact that mothers who use contraception typically have fewer children, which gives them more time and resources to ensure that each child receives all of the required immunizations. In addition, mothers who use family planning techniques are more likely to interact with health professionals, especially those who offer immunization services, using contraceptives frequently corresponds with improved access to healthcare services.

Mothers who had PNC checkups after giving birth had a higher incidence of childhood vaccinations than women who did not. This finding is supported by a study done in Ethiopia [45], Nigeria [47], three West African countries [16], sub-Saharan Africa [12]. This could be explained by the fact that at PNC visits, healthcare professionals can remind mothers of upcoming vaccinations and administer those that may have been overlooked after delivery or the early years of childhood. Additionally, PNC visits provide an opportunity to increase adherence to vaccination regimens and emphasize the value of immunization.

Compared to children whose previous birth interval was less than 24 months, children whose previous birth interval was 24 months or above had a high coverage of childhood vaccinations. This finding is consistent with the results of previous studies in Ethiopia [14], Nigeria [53] and Africa [14]. The reason for this is that mothers have more time to focus on each child’s health, ensure that they receive their vaccinations on time, recover from previous pregnancies, receive medical care without feeling overburdened, adhere to immunization schedules, and manage resources effectively, all of which improve vaccination coverage and the overall health of the child.

Children who had a health card had a higher prevalence of childhood vaccinations than children who did not have a health card. This result is similar to previous finding in Ethiopia [14,17] and Northern Nigeria [50]. The possible explanation for this could be that health cards encourage parents to make sure their children obtain the required vaccinations by reminding them to get immunizations on time and by making it easier for them to access healthcare services. Furthermore, the health card offers a transparent record of their immunization history, which aids parents and medical professionals in scheduling and monitoring vaccinations.

Compared to mothers who gave birth to their child at home, mothers who gave birth at health facilities had a greater incidence of childhood vaccinations. This is finding is supported by a study done in Ethiopia [17], Somali Region, Eastern Ethiopia [13], Nigeria [53], Northern Nigeria [50] and Sumatera, Indonesia [49]. This could be due to the reason that mothers who give birth in health facilities are more likely to get education from health professionals on the need of immunizations and the required immunization schedule. Additionally, babies delivered in health facilities are more likely to receive the recommended immunizations at birth, which include the polio, hepatitis B, and BCG vaccines. Besides, those who reported that distance to a health facility is not a significant problem had a higher incidence of childhood immunization compared to those who viewed distance as a major problem. This finding was in agreement with a study done in Somali Region, Eastern Ethiopia [13] and Nigeria [53].

The prevalence of childhood vaccinations was higher among children who received vitamin A than among children who did not receive it. This finding is consistent with studies in Ethiopia [14] and Nigeria [53]. This could be explained by the fact that mothers who took vitamin A supplements had better access to healthcare services, more health awareness, more frequent clinic visits, involvement in maternal and child health programs, a better perception of child health, and community influence that encouraged proactive health behaviors.

Compared to mothers who did not receive tetanus injections before birth, mothers who had received tetanus injections had a greater prevalence of childhood immunization. This result is consistent with studies conducted in Ethiopia [17]. This could be explained by the fact that mothers who were vaccinated against tetanus prior to delivery had more access to healthcare, experienced more frequent encounters with healthcare professionals, and were more cognizant of the significance of immunizations. Furthermore, they were exposed to vaccine information and professional encouragement through their involvement in prenatal care programs, which reaffirmed the need of adhering to recommended vaccination schedules for their children.

The geographical region of SSA had a significant impact on the incidence of childhood immunization. Children in Western and Southern SSA had higher incidence of childhood immunization than children in Eastern SSA. This finding was in line with those of other similar studies in Bangladesh [48] and SSA [12]. This could be due to differences in regional health policies, economic circumstances, cultural customs, or other factors like political unrest, conflict, or inadequate healthcare facilities.

## Conclusion

SSA had a low incidence of childhood vaccination and significant disparities. The results showed that the incidence of childhood immunizations varies significantly across the SSA region and countries. Incidence of childhood immunization was significantly associated with maternal education, occupation, age of mother, father education, media exposure, distance to health facilities, number of ANC visits, PNC, residence, place of delivery, received vitamin A1, contraceptive use, family size, wealth index, tetanus injections before birth, presence of health card, previous birth interval and SSA region. Therefore, public health initiatives that target low-income households, rural mothers, uneducated parents, and those who have not used maternal health care facilities should therefore increase the prevalence of childhood immunizations in order to enhance the health of children. The government and other stakeholders should properly endeavor to increase the incidence of childhood immunization by improving maternal tetanus injection, vitamin A supplementation, ANC and PNC visits, and institutional delivery. Furthermore, it is imperative to create policies and initiatives that tackle regional and national variations in childhood immunization rates and to actively work toward their implementation.

## Abbreviations

AIC: Akaike’s information criterion
ANC: Antenatal Care
AOR: Adjusted odds ratio
CI: Confidence intervals
DIC: Deviance information criterion
EAs: Enumeration areas
DHS: Demographic and Health Survey
ICC: Intra-cluster correlation
IRR: Incidence of Relative Risk
HP: Hurdle Poisson
HNB: Hurdle Negative Binomial
PNC: Postnatal Care
SSA: Sub-Saharan Africa
NB: Negative Binomial
ZIP: Zero-Inflated Poisson
ZINB: Zero-Inflated Negative Binomial
WHO: World Health Organization
