# Supplementary material for: Magnitude of childhood immunization and its associated factors in Sub-Saharan Africa: A mixed effect count regression analysis
Source: PLoS One. 2026 Aug 26;21(8):e0354918. doi: 10.1371/journal.pone.0354918 (PMC13514069; doi:10.1371/journal.pone.0354918)
Supplement: Supplementary file 2 [file pone.0354918.s003.docx]

Firstly, we sincerely thank the editor and reviewers for their thoughtful and constructive comments. Their insights have greatly helped us improve the clarity and quality of the manuscript. We have carefully addressed all suggestions, and detailed responses are provided below.

**Editors Comment**

**Comment:** Please ensure that your manuscript meets PLOS ONE's style requirements, including those for file naming. The PLOS ONE style templates can be found at <https://journals.plos.org/plosone/s/file?id=wjVg/PLOSOne_formatting_sample_main_body.pdf> and [https://journals.plos.org/plosone/s/file?id=ba62/PLOSOne_formatting_sample_title_authors_affiliations.pdf](https://track.editorialmanager.com/CL0/https:%2F%2Fjournals.plos.org%2Fplosone%2Fs%2Ffile%3Fid=ba62%2FPLOSOne_formatting_sample_title_authors_affiliations.pdf/1/010f019a4e17f3c3-38ad6257-d1ae-4d30-b8b2-a00865331be5-000000/vmqDTDXxb_86i4qoNOfvDp7HUzG49YDCV9E-mRDItvs=235)

**Response:** We have revised the manuscript according to PLOS ONE formatting guidelines and updated all file names to meet PLOS standards.

**Comment:** Thank you for uploading your study's underlying data set. Unfortunately, the repository you have noted in your Data Availability statement does not qualify as an acceptable data repository according to PLOS's standards. At this time, please upload the minimal data set necessary to replicate your study's findings to a stable, public repository (such as figshare or Dryad) and provide us with the relevant URLs, DOIs, or accession numbers that may be used to access these data. For a list of recommended repositories and additional information on PLOS standards for data deposition, please see [https://journals.plos.org/plosone/s/recommended-repositories](https://track.editorialmanager.com/CL0/https:%2F%2Fjournals.plos.org%2Fplosone%2Fs%2Frecommended-repositories/1/010f019a4e17f3c3-38ad6257-d1ae-4d30-b8b2-a00865331be5-000000/99RQ7OyfsV5VoSYNY1FEfRvxjYcGFncJQRR_HdhFDXA=235).

**Response:** Our study is based on **secondary data from the Demographic and Health Surveys (DHS)**, which are publicly available but cannot be redistributed by authors. In compliance with DHS data-use policy, we cannot upload the dataset to another repository. However, the data are freely accessible to all researchers upon registration through the DHS Program website. We have revised the Data Availability statement to include the correct access link and instructions for obtaining the data.

**Comment:** If the reviewer comments include a recommendation to cite specific previously published works, please review and evaluate these publications to determine whether they are relevant and should be cited. There is no requirement to cite these works unless the editor has indicated otherwise.

**Response:** We reviewed all suggested citations. Relevant works have been incorporated into the manuscript, while those not directly related to our study objectives were not added. Explanations are provided in the reviewer response.

Comment: please review your reference list to ensure that it is complete and correct. If you have cited papers that have been retracted, please include the rationale for doing so in the manuscript text, or remove these references and replace them with relevant current references. Any changes to the reference list should be mentioned in the rebuttal letter that accompanies your revised manuscript. If you need to cite a retracted article, indicate the article’s retracted status in the References list and also include a citation and full reference for the retraction notice.

**Response:** We reviewed the reference list for accuracy, completeness, and retraction status. Necessary corrections were made, and these changes are noted in the rebuttal.

**Reviewer 1**

**Comment 1:** The manuscript title is appropriate and on an important public health issue. It will contribute to knowledge.

**Response:** Thank you very much for your positive feedback. We appreciate your recognition that the study addresses an important public health issue and contributes valuable knowledge to the field.

**Comment 2:** The abstract is well structured and content appropriate. A minor grammatical error needs to be corrected.

**Response:** Thank you for your observation. We have carefully reviewed and corrected the minor grammatical error in the abstract to ensure clarity and accuracy.

**Comment 3:** The introduction provides the statement of the problem, magnitude of the problem, and objectives of the study.

**Response:** We are grateful for your positive assessment of the Introduction section. We are pleased that it clearly presents the problem statement, magnitude, and study objectives as intended.

**Comment 4:** The method section is well written and detailed.

**Response:** Thank you for acknowledging the quality and clarity of the Method section. We appreciate your recognition of our efforts to provide sufficient methodological detail for transparency and reproducibility.

**Comment 5:** Results are well presented.

**Response:** Thank you for your positive comment. We are glad that the presentation of our results was clear and well organized.

**Comment 6:** The study findings are well discussed and the conclusion is appropriate.
**Response:** We sincerely appreciate your encouraging feedback. We are pleased that the discussion and conclusion effectively interpret our findings and align with the study objectives.

**Reviewer 2**

**Comment:** This study is important because it covers a large geographical area and compares 33 countries simultaneously. It also contributes new cross-country comparisons and identifies significant predictors of childhood immunization in Africa.

**Response:** Thank you for recognizing the significance of our study. We appreciate your positive feedback. Our aim was indeed to provide a comprehensive cross-country analysis across 33 African countries to identify key predictors of childhood immunization. We are pleased that you found the broad geographical coverage and comparative approach valuable contributions to the existing literature

**Comment:** Data Source and Time Period: The study uses “the most recent Demographic and Health Surveys (DHS) data from 33 SSA countries between 2012 and 2022.” Since this period spans ten years, do you believe that meaningful comparisons are possible across such a wide time frame? Knowledge, attitudes, and practices regarding childhood immunization have improved considerably over the years. For example, increasing trends in immunization coverage are reported at https://doi.org/10.1371/journal.pgph.0004206. It is recommended to restrict the data range to surveys conducted within a 5-year window or, alternatively, include survey year as a covariate or random effect to control for temporal variability.

**Response:** Thank you for this important comment. We agree that using data collected over a 10-year period could introduce temporal variability, given the potential changes in knowledge, attitudes, and practices regarding childhood immunization over time. To address this concern, we included the survey year as an independent variable in our analysis to control for potential temporal effects. However, the survey year was not statistically significant in our models, suggesting that variation in immunization-related outcomes across survey years was minimal after adjusting for other covariates. Therefore, we retained the full dataset to preserve statistical power and maintain representation from all 33 SSA countries.

**Comment:** Operational Definition of the Dependent Variable: Please include a clear operational definition for the dependent variable, specifying that it represents the total of eight vaccine doses. Other studies categorize childhood immunization as including one dose of BCG, three doses of pentavalent DPT–HepB–Hib, three doses of PCV, three doses of OPV, two doses of Rota virus, and one dose of MCV1 (see https://doi.org/10.1186/s12199-021-00984-8 ).

**Response:** Thank you for this important comment. In our study, the dependent variable was defined in accordance with WHO guidelines and previous research (<https://doi.org/10.3402/gha.v9.30963>, <https://doi.org/10.1186/s41182-021-00319-x>, and <https://doi.org/10.1186/s13690-021-00581-9>) for children aged 12–23 months. Specifically, a child was considered fully immunized if they had received one dose of Bacillus Calmette–Guérin (BCG), three doses of diphtheria–pertussis–tetanus (DPT), three doses of polio vaccine (excluding the birth dose), and one dose of measles-containing vaccine (MCV1). Thus, the total represents eight essential vaccine doses required for a child to be classified as fully immunized.

**Comment:** Statistical Analysis Section: The statement “Logistic regression and survival analysis are two techniques for modeling such data after the count is converted to binary values [21, 32]” duplicates information already presented in the introduction. Moreover, since your main analytical approach is count regression, this section should emphasize the advantages of mixed-effect count regression (e.g., handling over dispersion, accounting for clustering, and managing zero-inflated data). Elaborating on why this model was preferred based on your data structure would strengthen the methodological justification.

**Response:** Thank you for this valuable comment. We have removed the duplicate statement, “Logistic regression and survival analysis are two techniques for modeling such data after the count is converted to binary values [21, 32],” from the Statistical Analysis section. In addition, we have expanded the section to provide a clearer justification for the use of mixed-effect count regression models. Specifically, we elaborated that this approach was chosen because it appropriately accounts for over-dispersion in the count data, addresses potential clustering of observations within survey clusters or countries, and effectively manages excess zeros in the outcome variable. These features make the mixed-effect count regression model well-suited to the hierarchical and overdispersed nature of our data.

**Comment:** Clarification Needed: The statement “The traditional count regression model’s application is inappropriate and reasonable since it assumes that there is no relationship between individual data [30]” is unclear. Please clarify what you mean — specifically, how the assumption of independence among individual observations affects model suitability.

**Response:** Thank you for this helpful comment. We have revised the statement for clarity. The traditional count regression model assumes that individual observations are independent of one another, meaning it does not account for potential dependencies or clustering within the data (for example, children nested within households or communities). However, our data have a hierarchical structure, where observations within the same cluster or country may be correlated. To address this, we employed a multilevel (mixed-effect) count regression model, which accounts for such dependencies by including random effects. We also compared the traditional count regression model with the multilevel count model using the likelihood ratio test (LRT), and the results indicated that the multilevel model provided a significantly better fit to the data.

**Comment:** Model Selection and Over dispersion Issue: Expanding the explanation of why MZIP was finally chosen if over-dispersion was detected (variance-to-mean ratio = 1.53), then a Negative Binomial–based approach might have been more appropriate than ZIP.

**Response:** Thank you for this valuable comment. You are correct that when over-dispersion is not primarily due to excess zeros, a Negative Binomial–based approach is often more appropriate than a Zero-Inflated Poisson (ZIP) model. In our analysis, we assessed over-dispersion using the Pearson chi-square ratio, likelihood ratio tests, and the Vuong test. The results indicated the presence of over-dispersion; however, this over-dispersion was mainly attributable to excess zeros in the data rather than heterogeneity in the count process. After accounting for these excess zeros through the mixed-effect zero-inflated Poisson (MZIP) model, the over-dispersion issue was no longer evident, confirming that MZIP was the most suitable model for our data structure.

**Comment:** Interpretation and Policy Relevance. The discussion should better link statistical results with public health implications. For example, highlight how maternal education, socioeconomic status, or healthcare accessibility influence vaccination uptake. The policy implications of these predictors should be explicitly discussed to enhance the paper’s applied relevance.

**Response:** Thank you for this insightful comment. We have revised the Discussion section to strengthen the connection between our statistical findings and their public health implications. Specifically, we now elaborate on how maternal education, socioeconomic status, and healthcare accessibility influence childhood immunization uptake. For instance, higher maternal education is linked to greater awareness and acceptance of vaccination services; children from wealthier households are more likely to complete immunization schedules due to reduced financial and access barriers; and proximity to healthcare facilities significantly improves vaccination coverage. We have also expanded the policy implications section to emphasize that improving maternal education, reducing socioeconomic inequalities, and enhancing access to primary healthcare—especially in rural and underserved areas—are critical strategies for increasing immunization coverage across sub-Saharan Africa.

**Comment:** Overall Recommendation The manuscript addresses an important topic and uses an appropriate analytical framework. However, before publication, the paper requires revisions to improve methodological clarity, consistency in model selection, and explicit operational definitions. Once these issues are addressed, the study has strong potential to contribute valuable evidence to the literature on childhood immunization and health equity in Sub-Saharan Africa.

**Response:** Thank you very much for your thoughtful overall assessment and constructive feedback. We appreciate your recognition of the importance of our topic and the suitability of our analytical framework. In response to your recommendations, we have thoroughly revised the manuscript to enhance methodological clarity, ensure consistency in model selection and justification, and include explicit operational definitions of key variables. We believe these revisions have substantially strengthened the rigor and clarity of our study. We are confident that the revised manuscript now provides a clearer and more comprehensive contribution to the literature on childhood immunization and health equity in sub-Saharan Africa.

Thanks for all.

Setegn Muche Fenta

Corresponding Authors
