# Supplementary material for: Magnitude of childhood immunization and its associated factors in Sub-Saharan Africa: A mixed effect count regression analysis
Source: PLoS One. 2026 Aug 26;21(8):e0354918. doi: 10.1371/journal.pone.0354918 (PMC13514069; doi:10.1371/journal.pone.0354918)
Supplement: Peer review history

# Peer review: Decision Letter 0

**DOI:** 10.1371/journal.pone.0354918.r001

**Authors:** Omar Enzo Santangelo

4 Nov 2025

Dear Dr. Fenta,

Thank you for submitting your manuscript to PLOS ONE. After careful consideration, we feel that it has merit but does not fully meet PLOS ONE’s publication criteria as it currently stands. Therefore, we invite you to submit a revised version of the manuscript that addresses the points raised during the review process.

==============================

**ACADEMIC EDITOR:**

==============================

Please submit your revised manuscript by Dec 19 2025 11:59PM. If you will need more time than this to complete your revisions, please reply to this message or contact the journal office at plosone@plos.org. When you're ready to submit your revision, log on to https://www.editorialmanager.com/pone/ and select the 'Submissions Needing Revision' folder to locate your manuscript file.

- A rebuttal letter that responds to each point raised by the academic editor and reviewer(s). You should upload this letter as a separate file labeled 'Response to Reviewers'.
- A marked-up copy of your manuscript that highlights changes made to the original version. You should upload this as a separate file labeled 'Revised Manuscript with Track Changes'.
- An unmarked version of your revised paper without tracked changes. You should upload this as a separate file labeled 'Manuscript'.

If applicable, we recommend that you deposit your laboratory protocols in protocols.io to enhance the reproducibility of your results. Protocols.io assigns your protocol its own identifier (DOI) so that it can be cited independently in the future. For instructions see: https://journals.plos.org/plosone/s/submission-guidelines#loc-laboratory-protocols. Additionally, PLOS ONE offers an option for publishing peer-reviewed Lab Protocol articles, which describe protocols hosted on protocols.io. Read more information on sharing protocols at https://plos.org/protocols?utm_medium=editorial-email&utm_source=authorletters&utm_campaign=protocols.

We look forward to receiving your revised manuscript.

Kind regards,

Omar Enzo Santangelo

Academic Editor

PLOS ONE

Journal Requirements:

When submitting your revision, we need you to address these additional requirements.

1. Please ensure that your manuscript meets PLOS ONE's style requirements, including those for file naming. The PLOS ONE style templates can be found at

https://journals.plos.org/plosone/s/file?id=wjVg/PLOSOne_formatting_sample_main_body.pdf and

https://journals.plos.org/plosone/s/file?id=ba62/PLOSOne_formatting_sample_title_authors_affiliations.pdf

2. Thank you for uploading your study's underlying data set. Unfortunately, the repository you have noted in your Data Availability statement does not qualify as an acceptable data repository according to PLOS's standards.

At this time, please upload the minimal data set necessary to replicate your study's findings to a stable, public repository (such as figshare or Dryad) and provide us with the relevant URLs, DOIs, or accession numbers that may be used to access these data. For a list of recommended repositories and additional information on PLOS standards for data deposition, please see https://journals.plos.org/plosone/s/recommended-repositories.

3. If the reviewer comments include a recommendation to cite specific previously published works, please review and evaluate these publications to determine whether they are relevant and should be cited. There is no requirement to cite these works unless the editor has indicated otherwise.

4. Please review your reference list to ensure that it is complete and correct. If you have cited papers that have been retracted, please include the rationale for doing so in the manuscript text, or remove these references and replace them with relevant current references. Any changes to the reference list should be mentioned in the rebuttal letter that accompanies your revised manuscript. If you need to cite a retracted article, indicate the article’s retracted status in the References list and also include a citation and full reference for the retraction notice.

[Note: HTML markup is below. Please do not edit.]

Reviewers' comments:

Reviewer's Responses to Questions

**Comments to the Author**

1. Is the manuscript technically sound, and do the data support the conclusions?

Reviewer #1: Yes

Reviewer #2: Partly

**********

2. Has the statistical analysis been performed appropriately and rigorously?

Reviewer #1: Yes

Reviewer #2: Yes

**********

3. Have the authors made all data underlying the findings in their manuscript fully available?

The PLOS Data policy

Reviewer #1: Yes

Reviewer #2: No

**********

4. Is the manuscript presented in an intelligible fashion and written in standard English?

Reviewer #1: Yes

Reviewer #2: Yes

**********

Reviewer #1: The manuscript is title is appropriate and on an important public health issue. It will contribute to knowledge.

The abstract is well structured and content appropriate. A minor grammatical error needs to be corrected.

The introduction provides the statement of the problem, magnitude of the problem and objectives of the study.

The method section is well written and detailed.

Results are well presented

The study findings are well discussed and the conclusion is appropriate

Reviewer #2: Reviewer Comments on the Article

Title: Magnitude of Childhood Immunization and Its Associated Factors in Sub-Saharan Africa: A Mixed-Effect Count Regression Analysis

This study is important because it covers a large geographical area and compares 33 countries simultaneously. It also contributes new cross-country comparisons and identifies significant predictors of childhood immunization in Africa. However, I would like to raise the following comments and questions:

1. Data Source and Time Period:

The study uses “the most recent Demographic and Health Surveys (DHS) data from 33 SSA countries between 2012 and 2022.” Since this period spans ten years, do you believe that meaningful comparisons are possible across such a wide time frame? Knowledge, attitudes, and practices regarding childhood immunization have improved considerably over the years. For example, increasing trends in immunization coverage are reported at https://doi.org/10.1371/journal.pgph.0004206.

It is recommended to restrict the data range to surveys conducted within a 5-year window or, alternatively, include survey year as a covariate or random effect to control for temporal variability.

2. Operational Definition of the Dependent Variable:

Please include a clear operational definition for the dependent variable, specifying that it represents the total of eight vaccine doses. Other studies categorize childhood immunization as including one dose of BCG, three doses of pentavalent DPT–HepB–Hib, three doses of PCV, three doses of OPV, two doses of Rota virus, and one dose of MCV1 (see https://doi.org/10.1186/s12199-021-00984-8).

3. Statistical Analysis Section:

The statement “Logistic regression and survival analysis are two techniques for modeling such data after the count is converted to binary values [21, 32]” duplicates information already presented in the introduction. Moreover, since your main analytical approach is count regression, this section should emphasize the advantages of mixed-effect count regression (e.g., handling over dispersion, accounting for clustering, and managing zero-inflated data).

Elaborating on why this model was preferred based on your data structure would strengthen the methodological justification.

4. Clarification Needed:

The statement “The traditional count regression model’s application is inappropriate and reasonable since it assumes that there is no relationship between individual data [30]” is unclear. Please clarify what you mean — specifically, how the assumption of independence among individual observations affects model suitability.

5. Model Selection and Over dispersion Issue:

Expanding the explanation of why MZIP was finally chosen if overdispersion was detected (variance-to-mean ratio = 1.53), then a Negative Binomial–based approach might have been more appropriate than ZIP.

6. Interpretation and Policy Relevance

The discussion should better link statistical results with public health implications. For example, highlight how maternal education, socioeconomic status, or healthcare accessibility influence vaccination uptake. The policy implications of these predictors should be explicitly discussed to enhance the paper’s applied relevance.

Overall Recommendation

The manuscript addresses an important topic and uses an appropriate analytical framework. However, before publication, the paper requires revisions to improve methodological clarity, consistency in model selection, and explicit operational definitions. Once these issues are addressed, the study has strong potential to contribute valuable evidence to the literature on childhood immunization and health equity in Sub-Saharan Africa.

**********

what does this mean?). If published, this will include your full peer review and any attached files.

If you choose “no”, your identity will remain anonymous but your review may still be made public.

**Do you want your identity to be public for this peer review?**  For information about this choice, including consent withdrawal, please see our Privacy Policy

Reviewer #1: **Yes:** Prof. Tanimola Makanjuola Akande

Reviewer #2: **Yes:** Alebachew Taye Belay

**********

[NOTE: If reviewer comments were submitted as an attachment file, they will be attached to this email and accessible via the submission site. Please log into your account, locate the manuscript record, and check for the action link "View Attachments". If this link does not appear, there are no attachment files.]

To ensure your figures meet our technical requirements, please review our figure guidelines: https://journals.plos.org/plosone/s/figures

You may also use PLOS’s free figure tool, NAAS, to help you prepare publication quality figures: https://journals.plos.org/plosone/s/figures#loc-tools-for-figure-preparation.

NAAS will assess whether your figures meet our technical requirements by comparing each figure against our figure specifications.

Attachment Submitted filename: PONE-D-25-19476_reviewer1.pdf

# Author comment: Author response to Decision Letter 1

**DOI:** 10.1371/journal.pone.0354918.r002

19 Nov 2025

I have no comment. I am to say thanks you both reviewer and editor for important and contractive comment.

Attachment Submitted filename: Response to Reviewers.docx

# Peer review: Decision Letter 1

**DOI:** 10.1371/journal.pone.0354918.r003

**Authors:** Omar Enzo Santangelo

14 Jul 2026

Magnitude of childhood immunization and its associated factors in Sub-Saharan Africa: A mixed effect count regression analysis

PONE-D-25-19476R1

Dear Dr. Fenta,

We’re pleased to inform you that your manuscript has been judged scientifically suitable for publication and will be formally accepted for publication once it meets all outstanding technical requirements.

Within one week, you’ll receive an e-mail detailing the required amendments. When these have been addressed, you’ll receive a formal acceptance letter and your manuscript will be scheduled for publication.

An invoice will be generated when your article is formally accepted. Please note, if your institution has a publishing partnership with PLOS and your article meets the relevant criteria, all or part of your publication costs will be covered. Please make sure your user information is up-to-date by logging into Editorial Manager at Editorial Manager® and clicking the ‘Update My Information' link at the top of the page. For questions related to billing, please contact billing support.

If your institution or institutions have a press office, please notify them about your upcoming paper to help maximize its impact. If they’ll be preparing press materials, please inform our press team as soon as possible -- no later than 48 hours after receiving the formal acceptance. Your manuscript will remain under strict press embargo until 2 pm Eastern Time on the date of publication. For more information, please contact onepress@plos.org.

Kind regards,

Omar Enzo Santangelo

Academic Editor

PLOS One

Additional Editor Comments (optional):

Reviewers' comments:

Reviewer's Responses to Questions

**Comments to the Author**

Reviewer #2: All comments have been addressed

**********

2. Is the manuscript technically sound, and do the data support the conclusions?

Reviewer #2: Yes

**********

3. Has the statistical analysis been performed appropriately and rigorously?

Reviewer #2: Yes

**********

4. Have the authors made all data underlying the findings in their manuscript fully available?

The PLOS Data policy

Reviewer #2: Yes

**********

5. Is the manuscript presented in an intelligible fashion and written in standard English?

Reviewer #2: Yes

**********

Reviewer #2: The authors are thanked for their careful and constructive responses to all reviewer comments. All previously raised concerns have been adequately addressed, and the revisions have significantly improved the clarity, methodological rigor, and overall quality of the manuscript. I have no further concerns regarding research ethics, publication ethics, or dual publication.

**********

what does this mean?). If published, this will include your full peer review and any attached files.

If you choose “no”, your identity will remain anonymous but your review may still be made public.

**Do you want your identity to be public for this peer review?**  For information about this choice, including consent withdrawal, please see our Privacy Policy

Reviewer #2: No

**********

# Editor report: Acceptance letter

**DOI:** 10.1371/journal.pone.0354918.r004

**Authors:** Omar Enzo Santangelo

PONE-D-25-19476R1

PLOS One

Dear Dr. Fenta,

I'm pleased to inform you that your manuscript has been deemed suitable for publication in PLOS One. Congratulations! Your manuscript is now being handed over to our production team.

At this stage, our production department will prepare your paper for publication. This includes ensuring the following:

* All references, tables, and figures are properly cited

* All relevant supporting information is included in the manuscript submission,

* There are no issues that prevent the paper from being properly typeset

You will receive further instructions from the production team, including instructions on how to review your proof when it is ready. Please keep in mind that we are working through a large volume of accepted articles, so please give us a few days to review your paper and let you know the next and final steps.

Lastly, if your institution or institutions have a press office, please let them know about your upcoming paper now to help maximize its impact. If they'll be preparing press materials, please inform our press team within the next 48 hours. Your manuscript will remain under strict press embargo until 2 pm Eastern Time on the date of publication. For more information, please contact onepress@plos.org.

You will receive an invoice from PLOS for your publication fee after your manuscript has reached the completed accept phase. If you receive an email requesting payment before acceptance or for any other service, this may be a phishing scheme. Learn how to identify phishing emails and protect your accounts at https://explore.plos.org/phishing.

If we can help with anything else, please email us at customercare@plos.org.

Thank you for submitting your work to PLOS One and supporting open access.

Kind regards,

PLOS ONE Editorial Office Staff

on behalf of

Dr. Omar Enzo Santangelo

Academic Editor

PLOS One
